# Supplementary material for: Reduced Expression of PRX2/ATPRX1, PRX8, PRX35, and PRX73 Affects Cell Elongation, Vegetative Growth, and Vasculature Structures in Arabidopsis thaliana
Source: Plants (Basel). 2022 Dec 2;11(23):3353. doi: 10.3390/plants11233353 (PMC9740967; doi:10.3390/plants11233353)
Supplement: Supplementary file 1 [file plants-11-03353-s001.zip › plants-2013393-supplementary.pdf]

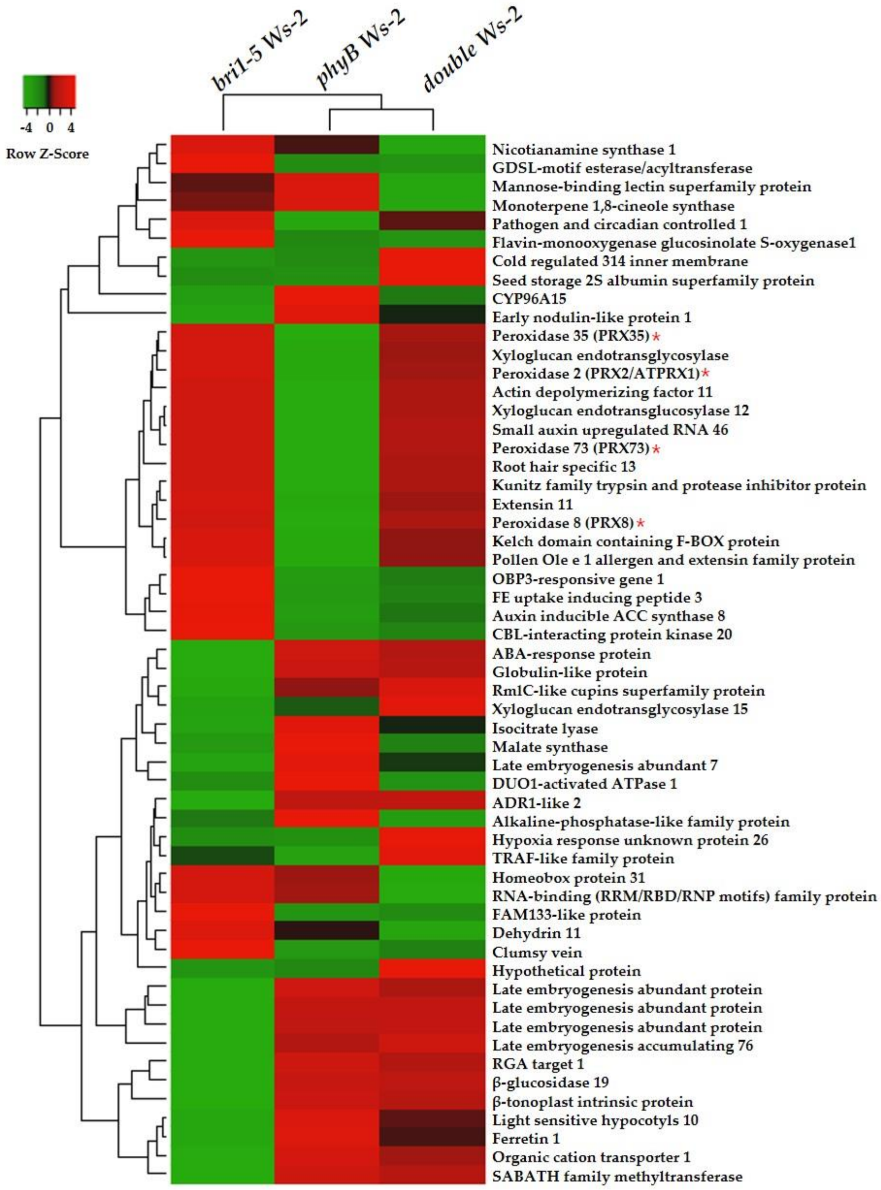

Figure S1. Identification of *PRX2*/*ATPRX1*, *PRX8*, *PRX35*, and *PRX73* genes among 56 genes co-regulated by brassinosteroids and light. The hierarchical cluster heat map shows the relative expression of genes differentially expressed in *bri1-5*, *phyB*, and *bri1-5*/*phyB* mutants among the 624 co-regulated genes. The color scale from green to red corresponds to gene expression intensity, ranging from low to high, respectively.

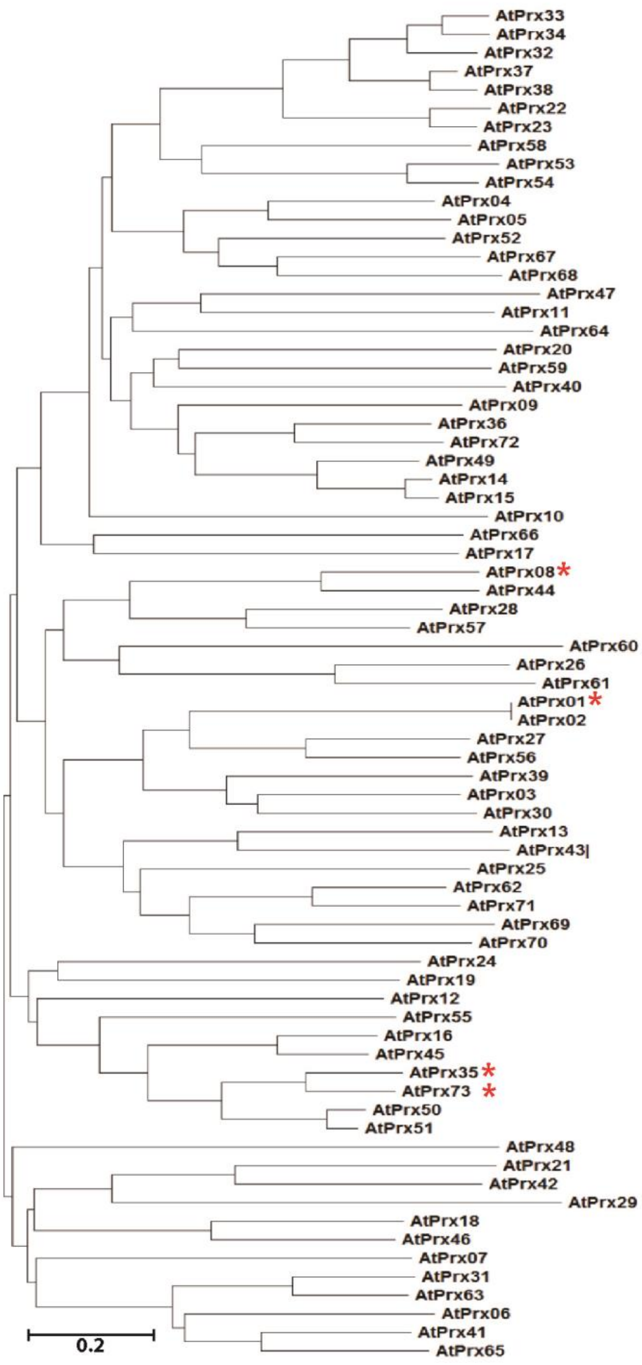

Figure S2. Phylogenetic tree based on the amino acid sequences of 73 *Arabidopsis* class III peroxidase (sourced from The Arabidopsis Information Resource) constructed by the neighbor-joining method in MEGA-X. Asterisks indicate four *PRX* genes investigated in this study.

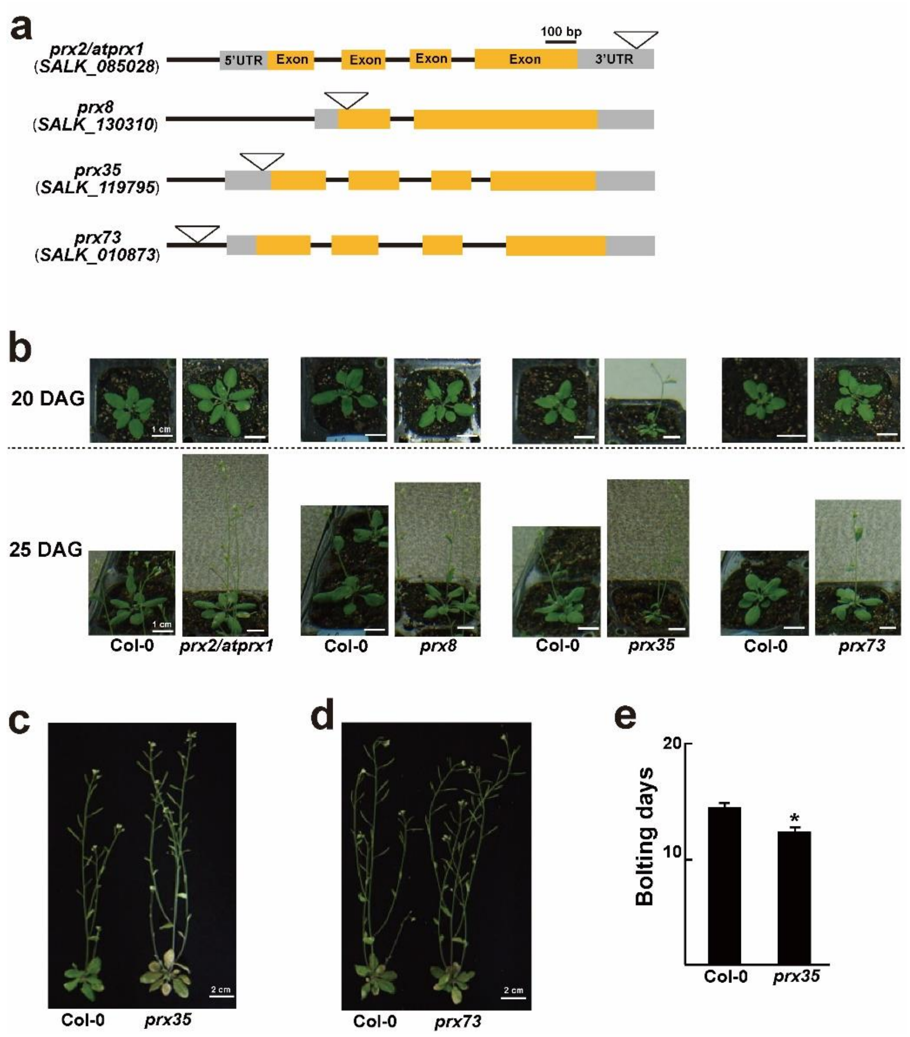

Figure S3. Characterization of *prx2/atprx1*, *prx8*, *prx35*, and *prx73* mutants. **a** Diagram of the genomic sequences of *PRX2/ATPRX1*, *PRX8*, *PRX35*, and *PRX73* showing the T-DNA insertion sites of the mutant lines *prx2/atprx1* (SALK_085028), *prx8* (SALK_130310), *prx35* (SALK_119795), and *prx73* (SALK_010873), respectively. Exons, introns, and untranslated regions are represented by orange boxes, black lines, and grey boxes, respectively. Open triangles indicate the positions of the T-DNA insertion. **b** Phenotypic comparisons among wild-type (Col-0) plants, and *prx2/atprx1*, *prx8*, *prx35*, and *prx73* mutants grown in soil for 20 and 25 days. Scale bar, 1 cm. **c, d** Phenotypic comparisons between wild-type (Col-0), *prx35* plants, and *prx73* plants grown in soil for 32 days. Scale bar, 2 cm. **e** Bolting days of wild-type (Col-0) and *prx35* plants ($n = 20$). The 10-day-old plants grown on the MS medium were transferred to soil. Asterisks indicate significant differences ($p < 0.001$, Student's *t*-test).

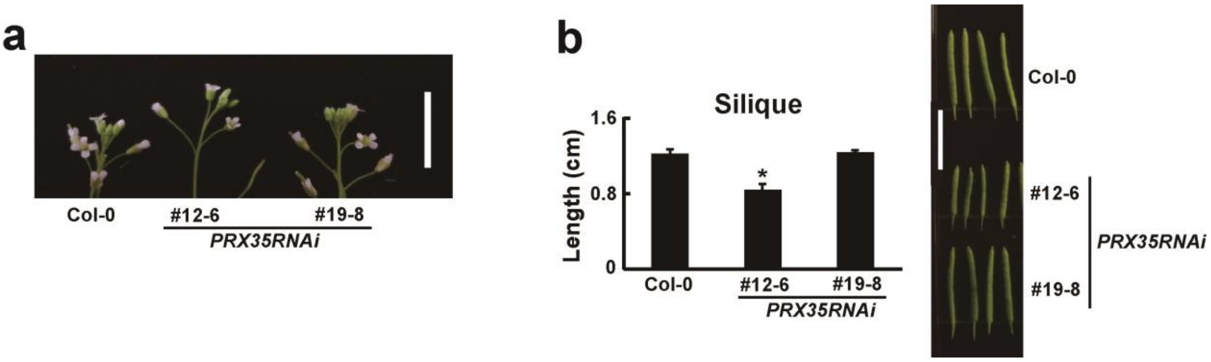

Figure S4. Morphological changes in reproductive organs of *PRX35RNAi* plants. **a** Inflorescence patterns of wild-type (Col-0) and *PRX35RNAi* plants grown for 6 weeks. Scale bar, 1 cm. **b** Silique lengths of 6-week-old soil-grown wild-type (Col-0) and *PRX35RNAi* plants ($n \geq 10$). Scale bar, 1 cm. Asterisks indicate significant differences ($P < 0.005$, Student's *t*-test).

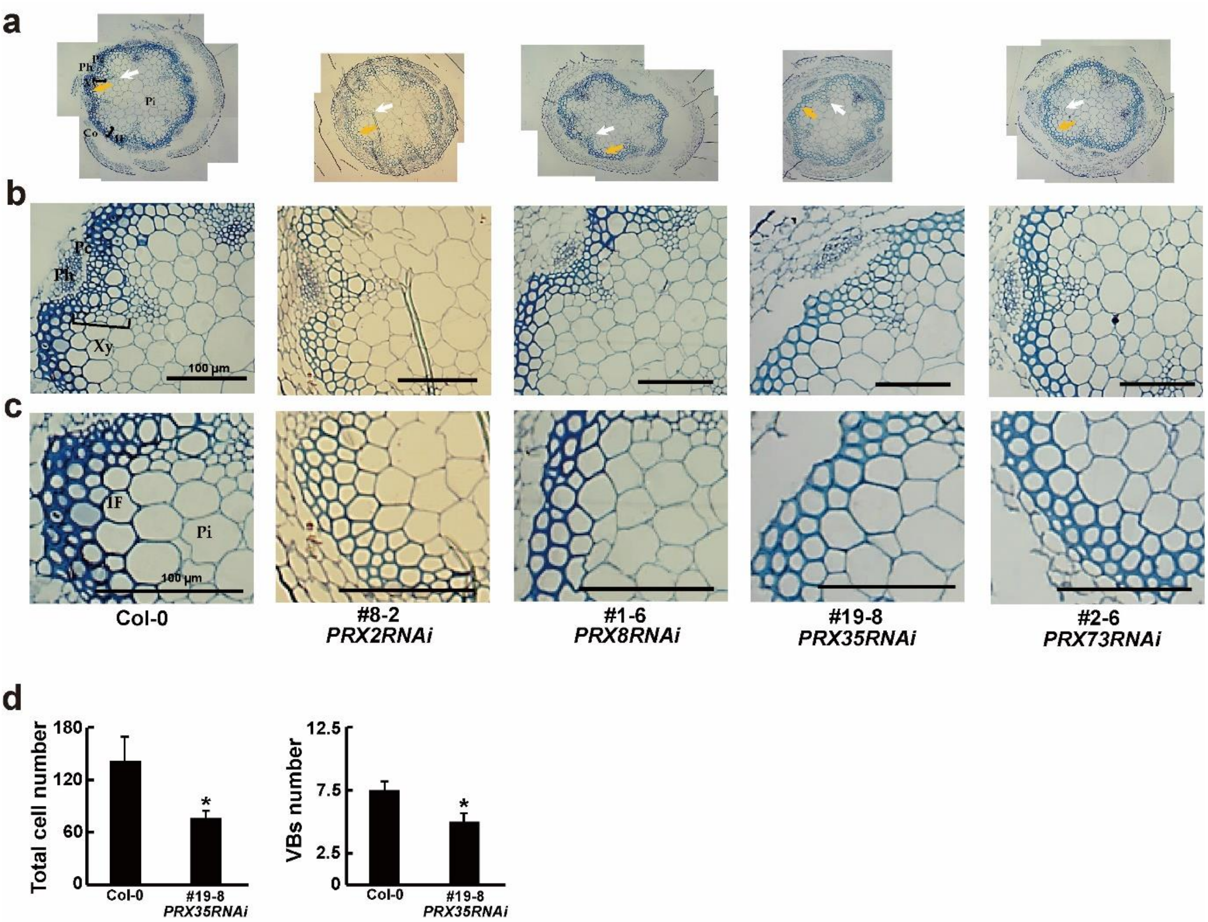

Figure S5. Transverse sections of basal main stems of wild-type (Col-0) plants and the independent *PRXsRNAi* lines for each RNAi construct. **a, b, c** Full (**a**) and enlarged photos (**b, c**) of the xylem and interfascicular fiber parts in toluidine blue-stained sections of resin-embedded stems of soil-grown 6-week-old wild-type (Col-0) and respective *PRXRNAi* plants. White and yellow arrows indicate the xylem and interfascicular fibers, respectively. The cortex (Co), phloem (Ph), xylem (Xy), pith (Pi), and interfascicular fiber (IF) are indicated. Scale bars, 100 μm. **d** Average total cell numbers of xylem cells and interfascicular fibers (left) along the vascular ring, and vascular bundles (right) in stems of soil-grown 6-week-old wild-type (Col-0) and *PRX35RNAi* (#19-8) plants. The values were the same in Figure 4. Error bars indicate the standard error of the mean ($n \geq 10$). Asterisks indicate significant differences ($P < 0.005$, Student's t-test).

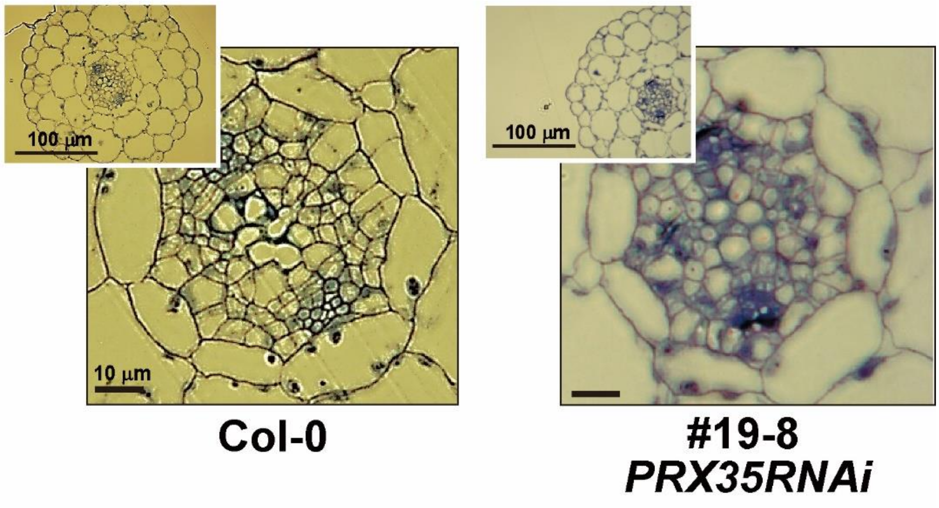

Figure S6. Transverse sections of hypocotyl and root regions in wild-type (Col-0) and *PRX35RNAi* seedlings. The photos of vascular bundles (upper left corner) in toluidine blue-stained sections of resin-embedded upper hypocotyls in wild-type (Col-0) and *PRX35RNAi* (#19-8) seedlings. Scale bars are presented in photos.

Table S1. Comparison of nucleotide sequences' identity within coding regions of *PRX2/ATPRX1*, *PRX8*, *PRX35*, *PRX44*, and *PRX73* genes

	PRX2/ATPRX1	*PRX8*	*PRX35*	*PRX44*	*PRX73*
PRX2/ATPRX1	-	*	76%	*	*
PRX8	*	-	75%	76%	*
PRX35	76%	75%	-	80%	80%
PRX44	*	76%	80%	-	*
PRX73	*	*	80%	*	-

*, No significant identity found. -, Alignment between same gene.

Table S2. Primers used for cloning of each *PRX* gene into the RNAi silencing Gateway vector

Gene ID	Name	Primer sequence (5′- 3′)
AT1G05250	*PRX2/ ATPRX1*	Prx2-F: CACCATGGCGATCAAGAACATTCTCGC
		Prx2-R: GTTAGGGAAGGCGCATCTCTTC
AT1G34510	*PRX8*	Prx8-F: CACCATGAGGGCAATCGCAGCTTGG
		Prx8-R: GTTGTTGAAGGCTCTGCAGTTTGT
AT3G49960	*PRX*35	Prx35-F: CACCATGGCTCGCTTCGATATTGTTC
		Prx35-R: GTTAAACGCACCACAATCACGACG
AT5G67400	*PRX*73	Prx73-F: CACCATGGCGCGGTTCAGTCTGGTT
		Prx73-R: GTTAAAGGCACCACAGTCACGAC

Table S3. Primers used for RT-qPCR amplification of four *PRX* genes

Gene ID	Name	Primer sequence (5′- 3′)
AT1G05250	*PRX2/ ATPRX1*	*prx2*R-F: CGAAATTGAACGATGCATTGCTAAA
		*prx2*R-R: CCATGTTCAGTGAGGTTCTGAAAT
AT1G34510	*PRX8*	*prx8*R-F: GGATCCTAAAATGGACAGCAAACTGA
		*prx8*R-R: AAATCTGACACAATCGACCTGGTTGAT
AT3G49960	*PRX*35	*prx35*R-F: CGAAGGGAACTTACCAGGACCTT
		*prx35*R-R: TTGTCAAACGTCTTGGGCGTGAC
AT5G67400	*PRX*73	*prx73*-F: CCGGACCAAATAACAAAGTTACAGAA
		prx73-R: GCGGTTACGAAAGCCTTGTTGAAA
AT4G05320	*UBQ10*	*UBQ10*-F: CCACCAAAGTTTTACATGAAACGAA
		UBQ10-R: TCCAGGACAAGGAAGGTATTCC

Table S4. Primers used for RT-qPCR amplification of lignin biosynthesis genes

Gene ID	Name	Primer sequence (5′- 3′)
AT2G37040	*PAL1*	PAL1-F: AAGATTGGAGCTTTCGAGGA
		PAL1-R: TCTGTTCCAAGCTCTTCCCT
AT2G30490	*C4H*	C4H-F: ACTGGCTTCAAGTCGGAGAT
		C4H-R: ACACGACGTTTCTCGTTCTG
AT1G51680	*4CL1*	4CL-F: TCAACCCGGTGAGATTTGTA
		4CL-R: TCGTCATCGATCAATCCAAT
AT5G48930	*HCT*	HCT-F: GCCTGCACCAAGTATGAAGA
		HCT-R: GACAGTGTTCCCATCCTCCT
AT2G40890	*C3H1*	C3H1-F: GTTGGACTTGACCGGATCTT
		C3H1-R: ATTAGAGGCGTTGGAGGATG
AT4G34050	*CCoAOMT1*	CCOAMT-F: CTCAGGGAAGTGACAGCAAA
		CCOAMT-F: GTGGCGAGAAGAGAGTAGCC
AT4G36220	*F5H1*	F5H-F: CTTCAACGTAGCGGATTTCA
		F5H-R: AGATCATTACGGGCCTTCAC
AT5G54160	*COMT*	COMT-F: TTCCATTGCTGCTCTTTGTC
		COMT-R: CATGGTGATTGTGGAATGGT
AT4G34230	*CAD*	CAD-F: TTGGCTGATTCGTTGGATTA
		CAD-R: ATCACTTTCCTCCCAAGCAT
AT4G05320	*UBQ10*	UBQ10-F: CCACCAAAGTTTTACATGAAACGAA
		UBQ10-R: TCCAGGACAAGGAAGGTATTCC

Table S5. Primers used for RT-qPCR amplification of lignin biosynthesis-regulation genes

Gene ID	Name	Primer sequence (5′ - 3′)
AT1G16490	*MYB58*	MYB58-F: CCAGAGAACAGAGCTCTTCAAGAG
		MYB58-R: ATGTATGAGGAGCTCGTAACTCTC
AT1G79180	*MYB63*	MYB63-F: GAACAGCTCAGGCTCAAGAGCAAC
		MYB63-R: ATGTATCATGAGCTCGTAGTTCTT
AT4G05320	*UBQ10*	UBQ10-F: CCACCAAAGTTTTACATGAAACGAA
		UBQ10-R: TCCAGGACAAGGAAGGTATTCC
